# Implications for annual retesting after a test-and-not-treat strategy for onchocerciasis elimination in areas co-endemic with *Loa loa* infection: an observational cohort study

**DOI:** 10.1016/S1473-3099(19)30554-7

**Published:** 2020-01

**Authors:** Sébastien DS Pion, Hugues Nana-Djeunga, Yannick Niamsi-Emalio, Cédric B Chesnais, Hugo Deléglise, Charles Mackenzie, Wilma Stolk, Daniel A Fletcher, Amy D Klion, Thomas B Nutman, Michel Boussinesq, Joseph Kamgno

**Affiliations:** a French Research Institute for Development—Unité Mixte Internationale 233 and French National Institute of Health and Medical Research—Unit 1175, University of Montpellier Montpellier, France; b Centre for Research on Filariasis and other Tropical Diseases, Yaounde, Cameroon; c Department of Pathobiology and Diagnostic Investigation, Michigan State University, East Lansing, MI, USA; d Department of Public Health, Erasmus University Medical Centre, Rotterdam, The Netherlands; e Department of Bioengineering and Biophysics Programme, University of California, Berkeley, CA, USA; f Laboratory of Parasitic Diseases, National Institute of Allergy and Infectious Diseases, National Institutes of Health, Bethesda, MD, USA

## Abstract

**Background:**

A test-and-not-treat (TaNT) strategy has been developed to prevent people with high concentrations of circulating *Loa loa* microfilariae (>20 000 microfilariae per mL) developing serious adverse events after ivermectin treatment during mass drug administration to eliminate onchocerciasis. An important question related to cost and programmatic issues is whether annual retesting is required for everyone. We therefore aimed to investigate changes in *L loa* microfilarial densities during TaNT campaigns run 18 months apart.

**Methods:**

In this observational cohort study, we assessed the participants of two TaNT campaigns for onchocerciasis. These campaigns, which were run by a research team, together with personnel from the Ministry of Health and community health workers, were done in six health areas (in 89 communities) in Okola health district (Cameroon); the first campaign was run between Aug 10, and Oct 29, 2015, and the second was run between March 7, and May 26, 2017. All individuals aged 5 years and older were invited to be screened for *Loa loa* microfilaraemia before being offered ivermectin (unless contraindicated). *L loa* microfilarial density was measured at the point of care using the LoaScope. All those with a *L loa* microfilarial density of 20 000 microfilariae per mL or less were offered treatment; in the first 2 weeks of the 2015 campaign, a higher exclusion threshold of 26 000 microfilariae per mL or less was used. At both rounds of the intervention, participants were registered with a paper form, in which personal information were collected. In 2017, we also recorded whether each individual reported participation in the 2015 campaign. The primary outcome, assessed in all participants, was whether *L loa* microfilarial density was above or below the exclusion threshold (ie, the criteria that guided the decision to treat).

**Findings:**

In the 2015 TaNT campaign, 26 415 people were censused versus 29 587 people in the 2017 TaNT campaign. All individuals aged 5 years and older without other contraindications to treatment (22 842 people in 2015 and 25 421 people in 2017) were invited to be screened for *L loa* microfilaraemia before being offered ivermectin. In 2015, 16 182 individuals were examined with the LoaScope, versus 18 697 individuals in the same communities in 2017. 344 (2·1%) individuals were excluded from ivermectin treatment because of a high *L loa* microfilarial density in 2015, versus 283 (1·5%) individuals in 2017 (p<0·0001). Records from 2017 could be matched to those from 2015 for 6983 individuals (43·2% of the 2015 participants). In this cohort, in 2017, 6981 (>99·9%) of 6983 individuals treated with ivermectin in 2015 had *L loa* microfilariae density below the level associated with neurological serious adverse events.

**Interpretation:**

Individuals treated with ivermectin do not need to be retested for *L loa* microfilaraemia before the next treatment, provided that they can be re-identified. This adjusted approach will enable substantial cost savings and facilitate reaching programmatic goals for elimination of onchocerciasis in areas that are co-endemic for loiasis.

**Funding:**

Bill & Melinda Gates Foundation, Division of Intramural Research (National Institute of Allergy and Infectious Diseases, US National Institutes of Health).

## Introduction

Loiasis, which is often referred to as the eye worm disease because of the subconjunctival migration of the adult worm seen in some infected individuals, is a vector-borne parasitic infection. Loiasis is endemic to Africa from South Sudan in the east to southeastern Benin in the west, and to the northern border of Zambia and Angola in the south.^1^ *Loa loa*, the filarial parasite responsible for loiasis, is transmitted between humans through the painful bites of female tabanids (deer flies), *Chrysops silacea* and *Chrysops dimidiata*. In endemic areas, loiasis transmission occurs throughout the year, with peaks during the rainy seasons,^2^, ^3^ and reinfection is common. Together with the long lifespan of adult worms—for instance, a female worm was removed from the eyelid of a patient 21 years after they visited a loiasis-endemic area^4^—this continuous transmission contributes to the chronicity of infection. Adult worms live in the upper layer of the epidermis and in the conjunctival tissues. After mating with male worms, female worms produce embryos or microfilariae that circulate in the peripheral bloodstream with a diurnal periodicity. Some infected individuals can clear microfilariae from their bloodstream, whereas others harbour tens to hundreds of thousands of microfilariae per mL of blood, a condition sometimes termed hypermicrofilaraemia. The reasons for this variability in response are not entirely understood but it could be due, in part, to a genetic predisposition.^5^, ^6^ However, the major problem associated with high-grade *L loa* microfilaraemia first emerged in the context of the large-scale treatment with ivermectin to combat onchocerciasis in central Africa during the early 1990s. In 1997, data showed that serious adverse events (SAEs), sometimes with fatal outcomes, could occur in people with high concentrations of circulating *L loa* microfilariae after a standard dose (150 μg/kg) of ivermectin.^7^ These SAEs appeared to be triggered by the rapid and massive death of the ivermectin-sensitive *L loa* microfilariae. Since then, implementation of ivermectin-based community treatment for onchocerciasis elimination has been halted or delayed in some foci of central Africa.

**Research in context**

**Evidence before this study**

In 2015, a large-scale evaluation of a test-and-not-treat strategy provided proof of concept that testing for high *Loa loa* microfilariae density before treatment prevents occurrence of severe adverse events after ivermectin treatment in areas that are co-endemic for onchocerciasis and loiasis. Although the strategy is considered promising, there is concern about the increased cost of this strategy relative to standard community-directed treatment with ivermectin. The cost difference over the programme lifespan is substantially dependent on whether people are retested every year. The objective of our study was to evaluate whether the *L loa* microfilariae density in a person not at risk of serious adverse events (those with a density of less than 20 000 microfilariae per mL) who received ivermectin increased above this threshold level 18 months after treatment. We searched PubMed and ISI Web of Knowledge with the search terms “(loiasis OR loase OR *Loa loa*) AND (ivermectin$) AND (trial OR therapeuti$)”, for work published in English or French before April 1, 2019. We also checked the references of all studies identified by these methods. A 2019 meta-analysis of previous trials of ivermectin on *L loa* microfilariae density indicated that, 1 year after treatment, none of the 238 individuals with an initial *L loa* microfilariae density of less than 20 000 microfilariae per mL were at risk of serious adverse events after ivermectin treatment. This finding suggested that, when eligible individuals are treated with ivermectin once, they can safely receive annual ivermectin treatment without testing.

**Added value of this study**

To our knowledge, our study is the largest to evaluate the development of individual *Loa loa* microfilarial density over two campaigns of community treatment with ivermectin for onchocerciasis. Our results suggest that individuals with an initial *L loa* microfilariae density of less than 20 000 microfilariae per mL who have received standard treatment with ivermectin as part of onchocerciasis elimination activities are unlikely (at an observed frequency of 0·03%) to have a microfilarial density associated with an increased risk of *L loa*-related serious adverse events if retreated within 18 months, and they could be treated again without being retested.

**Implications of all the available evidence**

Since 95% of participants can be safely treated with ivermectin during any given test-and-not-treat campaign, not having to repeat quantification of microfilarial density the following year would enable substantial savings in time and money.

Between August and October 2015, we selectively treated people with ivermectin in an area of Cameroon where onchocerciasis and loiasis are co-endemic.^8^ To prevent the occurrence of SAEs, we used a test-and-not-treat (TaNT) strategy: we quantified *L loa* microfilaraemia in all consenting residents of the Okola health district who were aged 5 years or older (n=16 182) at the point of care. All individuals with more than 20 000 *L loa* microfilariae per mL, who were thereby deemed at-risk for SAEs, and those with contraindications to ivermectin (pregnant or breastfeeding women or those with a serious acute or chronic concomitant illness) were excluded from ivermectin treatment, but they were offered a single oral dose of albendazole 400 mg (unless this treatment was also contraindicated) for intestinal deworming. Individuals with more than 20 000 *L loa* microfilariae per mL were revisited after the campaign, to ascertain their onchocerciasis status by use of the standard skin-snip method. Those who were infected with *Onchocerca volvulus* received a 5-week daily treatment regimen with doxycycline (100 mg; unless contraindicated).

During this ivermectin-based TaNT campaign for onchocerciasis elimination, 95·5% of participants tested for *L loa* received ivermectin, and only 2·1% of participants were excluded for *L loa* densities of greater than this risk threshold.^8^ No SAEs occurred in any of the treated population. Because several ivermectin treatment campaigns are needed to achieve onchocerciasis elimination, an important question related to cost and programmatic issues is whether annual testing of the whole population for *L loa* microfilariae density is required. More specifically, do previously treated individuals require repeat testing? Data previously collected from 238 individuals with an initial *L loa* microfilariae density of less than 20 000 microfilariae per mL indicated that none was at risk of SAEs 1 year after receiving ivermectin.^9^

During a second TaNT campaign that was run in Okola between March and May 2017 (around 18 months after the initial campaign), we aimed to investigate changes in *L loa* microfilarial densities after the first treatment in 2015, at the individual and community levels.

## Methods

### Study design and participants

In this observational cohort study, we assessed the participants of two TaNT campaigns for onchocerciasis. These campaigns, which were run by a research team, together with personnel from the Ministry of Health and community health workers, were done in six health areas (in 89 communities) in Okola health district (Cameroon); the first campaign^8^ was run between Aug 10, and Oct 29, 2015, and the second was run between March 7, and May 26, 2017. All individuals aged 5 years and older were invited to be screened for *L loa* microfilaraemia before being offered ivermectin (unless contraindicated).

Our study was authorised by the National Ethics Committee of Cameroon (number 2013/11/370/L/CNERSH/SP) and approved by the Division of Operational Research at the Ministry of Health (number D30–571/L/MINSANTE/SG/DROS/CRSPE/BBM). All volunteers provided written signed consent (or parental consent in the case of minors) before blood sampling and again before receiving treatment.

### Procedures

*L loa* microfilarial density was measured at the point of care using the LoaScope.^10^ All those with a *L loa* microfilarial density of 20 000 microfilariae per mL or less were offered treatment; in the first 2 weeks of the 2015 campaign, a higher exclusion threshold of 26 000 microfilariae per mL or less was used, as explained previously.^8^

The TaNT studies were not initially designed to provide longitudinal data. Nonetheless, at both rounds of the intervention, participants were registered with a paper form. Personal information about participants that was collected on this form included their full name, age, sex, phone number, and a household number assigned during an exhaustive census that was done a few weeks before the campaign. Our team did the censuses, with the help of community members from each village. In 2017, we also recorded whether each individual reported participation in the 2015 campaign.

All data collected on paper forms were entered into an electronic database using double-entry for quality control. An automated script was developed with Stata version 15.1 to identify discrepancies between the two series of data entries from a given year, and all discrepancies were resolved by reference to the paper form. The 2015 data were matched to those from 2017 by use of participant name, age, village of residence and, when available, the individual barcode assigned to them in 2015. We assumed that names of individuals who participated in both campaigns might have been spelt differently, and we used a semi-automated algorithm to generate lists of likely matches. All matches with a high degree of certainty were validated manually. When the data forms were inconclusive, we contacted participants by phone for confirmation of their participation in the TaNT campaigns. This approach allowed us to define a sample of individuals tested in both 2015 and 2017.

### Outcomes

The primary outcome, assessed in all participants, was wether their *L loa* microfilarial density was above or below the exclusion threshold (ie, the criteria that guided the decision to treat).

### Statistical analysis

Unless otherwise stated, geometric means were used as a measure of central tendency. The prevalence and concentration of *L loa* microfilariae per mL of blood in all individuals tested in 2015 were compared with those of individuals tested in 2017 by use of χ^2^ (for prevalence) or Mann-Whitney test (for intensity). For those participating in both campaigns, the McNemar's test and Student's *t* tests for paired samples were used to compare the prevalence and intensity of *L loa* microfilarial infection between 2015 and 2017. We also constructed transition matrices to represent the development of microfilarial densities on a semi-quantitative scale in the groups: 0, 1–100, 101–500, 501–2000, 2001–10 000, 10 001–20 000, and more than 20 000 microfilariae per mL. All statistical tests were performed with Stata version 15.1.

### Role of the funding source

The funders of the study had no role in the study design, data collection, data analysis, data interpretation, or writing of the report. The corresponding author had full access to all the data in the study and had final responsibility for the decision to submit for publication.

## Results

In the 2015 TaNT campaign, 26 415 people were censused versus 29 587 people in the 2017 TaNT campaign. All individuals aged 5 years and older without other contraindications to treatment (22 842 people in 2015 and 25 421 people in 2017) were invited to be screened for *L loa* microfilaraemia before being offered ivermectin. In 2015, 16 182 individuals were examined with the LoaScope, versus 18 697 individuals in the same communities in 2017. 344 (2·1%) individuals were excluded from ivermectin treatment because of a high *L loa* microfilarial density in 2015, versus 283 (1·5%) individuals in 2017 (p<0·0001; table 1). Records from 2017 could be matched to those from 2015 for 6983 individuals (43·2% of the 2015 participants).

**Table 1:** Participants' characteristics and frequencies of contraindications to ivermectin treatment in 2015 and 2017

		**General population**		**Sample (n=6983)**	
		2015 (n=16 182)	2017 (n=18 697)	2015	2017
Male to female sex ratio		0·93	1·00	0·97	0·97
Median age (IQR)		18 (11–42)	19 (11–40)	17 (10–47)	19 (12–49)
*Loa loa* microfilarial density, mf/mL (95% CI)					
	Arithmetic mean	1465·90 (1376·30–1553·49)	928·69 (861·89–995·48)	1426·62 (1295·41–1557·82)	712·64 (612·72–812·57)
	Geometric mean of positive counts	2825·0 (2660·84–2999·22)	1484·72 (1394·95–1580·27)	2550·30 (2336·56–2783·59)	1123·82 (1017·06–1241·78)
Contraindication, n (%)					
	None	15 458 (95·5%)	18 100 (96·8%)	6716 (96·2%)	6816 (97·6%)
	*Loa loa* density >20 000 mf/mL	344 (2·1%)	281 (1·5%)	134 (1·9%)	85 (1·2%)
	Pregnancy	165 (1·0%)	250 (1·3%)	61 (0·9%)	57 (0·8%)
	Ill health	215 (1·3%)	66 (0·4%)	72 (1·0%)	25 (0·4%)
Treated with ivermectin, n (%)		15 369 (95·0%)	17 994 (96·2%)	6692 (95·8%)	6798 (97·4%)
Refusals, n (%)		89 (0·5%)	104 (0·6%)	24 (0·3%)	16 (0·2%)

mf=microfilariae.

**Figure 1:**
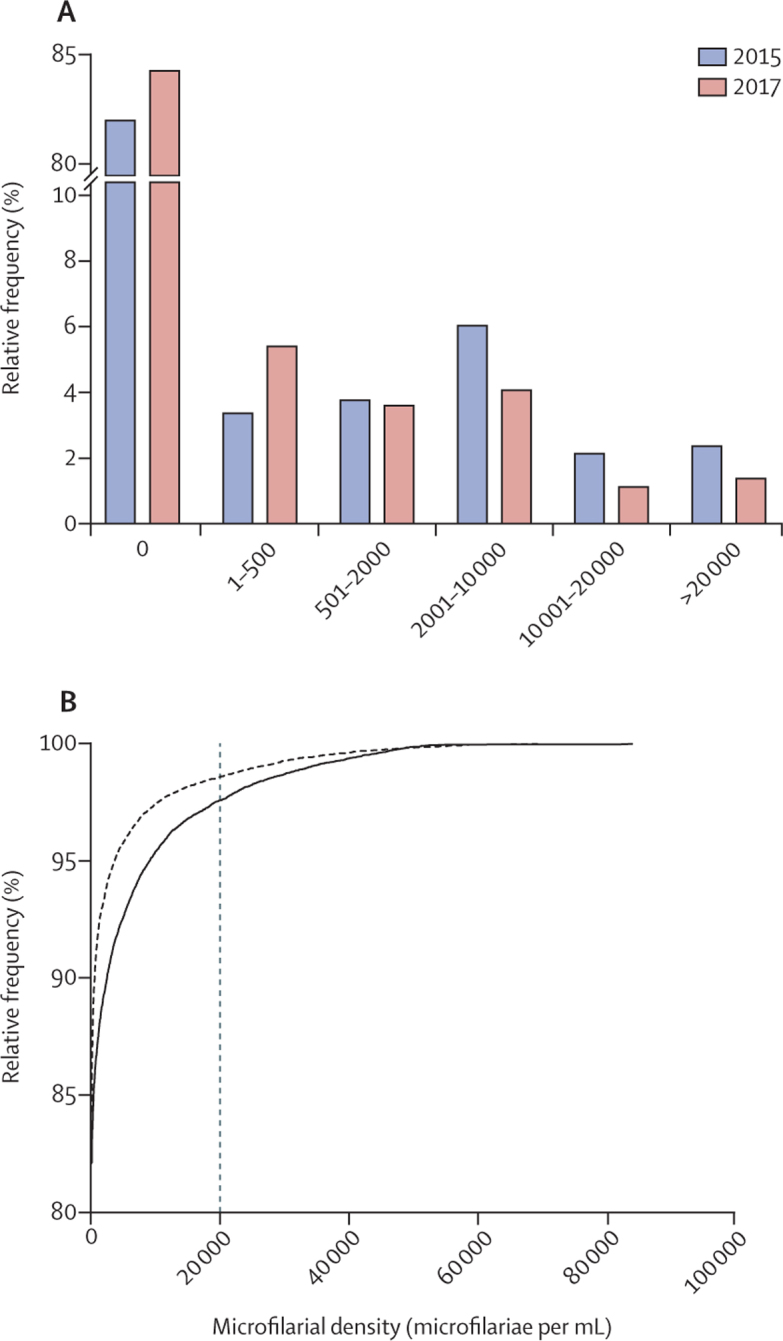
Frequency distribution (A) and cumulative frequency distribution (B) of *L loa* microfilarial density Data are in 16 182 individuals examined in 2015 (solid line) and in 18 697 individuals examined in 2017 (dotted line).

Based on the LoaScope results, the prevalence of *L loa* microfilaraemia decreased from 2015 to 2017: the condition was found in 2901 (17·9%) of 16 182 individuals in 2015, versus 2981 (15·9%) of 18 697 individuals in 2017 (p<0·0001). The geometric mean of positive *L loa* microfilarial densities decreased from 2825·00 microfilariae per mL (95% CI 2660·80–2999·22) in 2015, to 1484·72 microfilariae per mL (1394·95–1580·27) in 2017 (p<0·0001; table 1). This decrease in intensity of infection is reflective of a significant shift toward lower values in the frequency distribution of *L loa* microfilarial density (Mann-Whitney test, p<0·0001; figure 1).

The therapeutic coverage (people treated relative to the censused population) in the six health areas varied between 50·8% and 65·5% in 2015 and between 60·5% and 78·0% in 2017. Of the 6983 individuals whose *L loa* microfilarial density was assessed with the LoaScope in both 2015 and 2017, 6692 (95·8%) had received ivermectin in 2015, versus 6798 (97·4%) in 2017 (table 1). The prevalence of *L loa* microfilaraemia in these treated individuals decreased from 2015 to 2017: the condition was found in 1161 (17·3%) of 6692 individuals in 2015, versus 872 (13·0%) of 6692 individuals in 2017 (p<0·0001). The geometric mean of *L loa* microfilarial densities decreased from 2550·30 microfilariae per mL (95% CI 2336·56–2783·59) in 2015, to 1123·82 microfilariae per mL (1017·06–1241·78) in 2017 (p<0·0001). By contrast, the prevalence of microfilaraemia remained unchanged in those who were not treated with ivermectin in 2015 (present in 173 [59·5%] of 291 individuals in 2015 *vs* 173 [59·5%] of 291 individuals in 2017), although the geometric mean of microfilariae density decreased in these individuals, from 16 516·4 (13 170·0–20 713·1) in 2015, to 12 121·7 (9810·2–14 977·9) in 2017 (p<0·00001).

Among the 5531 individuals without detectable *L loa* microfilaraemia who received ivermectin in 2015, 5274 (95·4%) individuals had no detectable microfilaraemia in 2017, whereas 257 (4·6%) individuals developed microfilaraemia in 2017, but all of these individuals had microfilarial densities of less than 10 000 microfilariae per mL (table 2). Among the 1161 microfilaraemic individuals in 2015 who received ivermectin, in 2017, 957 (82·4%) individuals showed a reduced density, 160 (13·8%) showed a similar density, and 44 (3·8%) showed an increased density.

**Table 2:** Transition matrix of *Loa loa* microfilarial density in 2015 (left column) versus that in 2017 (top row), in individuals treated with ivermectin in 2015

	**0**	**1–100**	**101–500**	**501–2000**	**2001–10 000**	**10 000–20 000**	**>20 000**	**Total**
0	5274	11	201	38	7	0	0	5531
1–100	3	0	0	0	0	0	0	3
101–500	204	0	31	20	6	0	0	261
501–2000	179	1	54	40	14	0	0	288
2001–10 000	144	0	74	130	82	2	2	434
10 001–20 000	14	0	9	50	73	7	0	153
20 000–26 000	2	0	1	7	12	0	0	22
Total	5820	12	370	285	194	9	2	6692

Data are the number of individuals treated with ivermectin in 2015 within each recorded range of microfiliarial density (in microfilariae per mL) in 2015 versus that in 2017.

**Figure 2:**
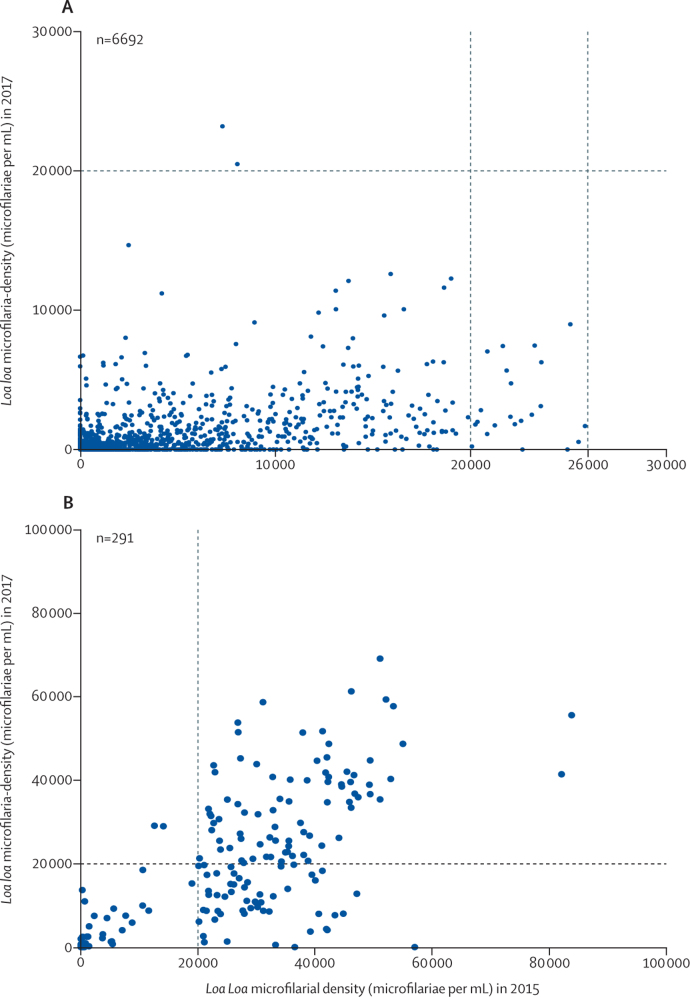
*Loa loa* microfilarial density in our sample of 6983 individuals examined in 2015 and 2017 (A) Individuals treated with ivermectin in 2015. (B) Individuals not treated with ivermectin in 2015.

Among the 118 individuals without detectable *L loa* microfilaraemia who were not treated with ivermectin in 2015, 109 (92·4%) individuals had no detectable microfilaraemia in 2017, and the remaining nine (7·6%) developed microfilaraemia in 2017; however, again, all of these individuals had microfilarial densities of less than 10 000 microfilariae per mL (table 3). Among the 173 individuals with microfilaraemia who were not treated with ivermectin in 2015, 67 (38·7%) individuals showed a reduced density, 94 (54·3%) showed a similar density, and 12 (7·0%) showed an increased density. Of the 134 individuals who did not receive ivermectin in 2015 because they had a *L loa* microfilarial density of more than 20 000 microfilariae per mL, 81 (60·4%) had a *L loa* microfilarial density of more than 20 000 microfilariae per mL in 2017. 53 (39·6%) individuals with no contraindications in 2017 had microfilarial densities below the risk threshold and received ivermectin without incident (figure 2; table 4). Two individuals (0·03% of ivermectin-treated individuals in 2015) had a microfilarial density slightly more than 20 000 microfilariae per mL in 2017 despite treatment: a man aged 75 years whose *L loa* density increased from 7294 microfilariae per mL to 23 208 microfilariae per mL, and a man aged 45 years whose *L loa* density increased from 8051 microfilariae per mL to 20 499 microfilariae per mL

**Table 3:** Transition matrix of *Loa loa* microfilarial density in 2015 (left column) versus that in 2017 (top row), in individuals not treated with ivermectin in 2015

	**0**	**1–100**	**101–500**	**501–2000**	**2001–10 000**	**10 000–20 000**	**>20 000**	**Total**
0	109	0	6	2	1	0	0	118
1–100	0	0	0	0	0	0	0	0
101–500	6	0	1	2	3	1	0	13
501–2000	1	0	1	2	3	1	0	8
2001–10 000	0	0	0	3	8	0	0	11
10 001–20 000	0	0	0	0	3	2	2	6
>20 000	2	0	0	3	19	29	81	134
Total	118	0	8	12	37	33	83	291

Data are the number of individuals not treated with ivermectin in 2015 within each recorded range of microfiliarial density (in microfilariae per mL) in 2015 versus that in 2017.

**Table 4:** Transition matrix of contraindications to ivermectin in the sample in 2015 (left column) versus that in 2017 (top row)

	**None**	***Loa loa* density >20 000 microfilariae per mL**	**Pregnancy**	**Ill health**	**Total**
None	6645	2	52	17	6716
*Loa loa* density >20 000 microfilariae per mL	53	81	0	0	134
Pregnancy	55	1	5	0	61
Ill health	63	1	0	8	72
Total	6816	85	57	25	6983

Data are number of participants.

Two individuals who did not receive ivermectin treatment in 2015 because of pregnancy (density of 14 105 microfilariae per mL) or ill health (12 569 microfilariae per mL) had a microfilarial density above the threshold 18 months later (28 999 microfilariae per mL and 29 109 microfilariae per mL).

6981 (>99·9%) of 6983 individuals treated with ivermectin in 2015 had a *L loa* microfilarial density below the exclusion threshold of 20 000 microfilariae per mL 18 months later.

## Discussion

The TaNT campaign that was run in the Okola health district in 2015 was successful in that more than 15 000 inhabitants of an area that is co-endemic for onchocerciasis and loiasis were treated with ivermectin without SAEs. A second TaNT campaign, with systematic testing for *L loa* microfilaraemia, was initially planned to take place 1 year later, to concur with the usual schedule of repeated community treatments with ivermectin against onchocerciasis. However, field activities were delayed by 6 months for logistical reasons, and the second round was therefore conducted 18 months after the first.

Assessment of *L loa* microfilarial density during this second campaign showed substantial impact of the first large-scale treatment on the microfilarial reservoir of *L loa*. The notable drop in *L loa* microfilarial density was expected to some degree, based on data from a 2019 literature review and meta-analysis^9^ of the effect of ivermectin on *L loa* microfilariae up to 1 year after treatment. However, our study indicates that the effect of ivermectin remains 18 months after the initial dose. A similar effect was seen in a community trial^11^ in an area neighbouring the Okola health district^,^ which found reductions in *L loa* microfilariae 1 year after ivermectin treatment that were comparable to those measured in our study 18 months after ivermectin distribution (appendix p 1).

The major finding of our study was that 99·97% of individuals treated with ivermectin in 2015 had a *L loa* microfilarial density below the exclusion threshold of 20 000 microfilariae per mL 18 months later. Together with the observation that no individuals with microfilarial densities of less than 20 000 microfilariae per mL who were treated with ivermectin in all previous trials^9^ (238 individuals) presented with a higher microfilarial count 1 year later, our findings suggest that individuals treated once with ivermectin could be safely retreated within 18 months without retesting. Although two individuals in our study who had microfilarial densities of less than 20 000 microfilariae per mL in 2015 had densities slightly greater than this value in 2017, it is important to recognise that the LoaScope threshold was deliberately chosen to be extremely conservative based on the currently available SAE data^7^, ^12^, ^13^, ^14^, ^15^ documenting neurological adverse events after ivermectin treatment only in individuals with densities of at least 50 000 microfilariae per mL. Thus, the two individuals whose counts increased to slightly greater than 20 000 microfilariae per mL in 2017 probably could have been safely treated with ivermectin without retesting.

Our study is unique in its scale and documentation of the stability and variability of *L loa* microfilarial density over time. *L loa* microfilarial density is generally considered to be very stable over time in untreated individuals.^16^, ^17^, ^18^ This general trend was confirmed in our study, in which approximately 70% of the participants (including amicrofilaraemic and microfilaraemic individuals) who did not receive ivermectin in 2015 had similar microfilarial densities 18 months later (ie, they remained in the same category in the transition matrix; table 3). Despite this finding, our data suggest that retesting of individuals who did not receive ivermectin in the previous round is important, since 51 (38·1%) of 134 individuals excluded from ivermectin treatment in 2015 because of high microfilarial densities had densities of 20 000 microfilariae per mL or less in 2017 and they could receive ivermectin, and two individuals, whose microfilarial density was below the threshold in 2015 but who were excluded because of pregnancy or ill health, had microfilarial densities that precluded ivermectin treatment in 2017. Among the ivermectin-treated individuals with microfilaraemia in 2015, most (82·4%) people had decreased counts in 2017; only 3·8% moved into a higher category in the transition matrix, and only two people had densities of more than 20 000 microfilariae per mL (table 2). Potential reasons for the variability in microfilarial densities include the diurnal periodicity of *L loa* microfilariae in the blood and resultant variation depending on the exact time of the blood draws; acquisition or attrition of fertile female worms; and alterations in host response after ivermectin treatment.^19^

The main limitation of our study was the relatively low number of individuals (only 43·2%) who could be definitively matched between the 2015 and 2017 campaigns. The main source of this problem was uncertainty surrounding the identity of the participants, due to no standardised spelling of certain names and inaccurate recording of age in the elderly population. Also, a high proportion of individuals participated in the initial TaNT campaign while visiting neighbouring villages (and thus could not be matched on the basis of village in 2017). Although every participant was given an individual card containing their name, phone number, result of *L loa* microfilarial density assessment, and treatment received, few people brought this card with them when they presented for retreatment in 2017. Despite this limitation, it is unlikely that individuals who participated in both rounds but whose records could not be definitively matched had a different response to ivermectin treatment than those who could be matched unequivocally, and the number of actual matched records (n=6983) was sufficient for statistical power and accuracy around the estimates.

Not having to retest everyone annually could have important implications for the costs and practical implementation of the strategy. In a neighbouring area of Cameroon, where the TaNT strategy was implemented in a pilot study^20^ by local health personnel and community volunteers (with oversight by the research team), the costs were estimated at US$9·2 per person tested. Notably, it was projected that, under programmatic implementation scenarios, these costs could be reduced to about $5 per person treated.^20^, ^21^ If participation is high during the first round, far fewer people will need to be tested during the subsequent round. Indeed, about 95% of the participants from round N would not have to be tested during round N + 1. The extra cost of the TaNT strategy during subsequent years is likely to be much smaller than in the first round. After several years of TaNT—the duration of which remains to be determined—ivermectin-naive people could be tested and treated in a central health structure (health area or health district) whereas the rest of the population could be treated using the classic community-directed treatment with ivermectin strategy. This approach would lead to dramatic cost savings (eg, less time spent by health personnel for testing, lower costs for LoaScopes and capillaries).

Additionally, previously treated individuals could be retreated even in the absence of a so-called LoaScopist (the volunteer in charge of testing the *L loa* microfilariae density with a LoaScope), providing more flexibility in the implementation (ie, how and when people are treated) and possibly an increase in the number of people treated per day. These benefits require that people treated in the previous round can be re-identified easily and reliably. Although identification could theoretically be done using the individual card (containing their name, phone number, result of *L loa* microfilarial density assessment, and treatment received), most people in our study did not present the card during the second campaign. Treatment registers specifically designed for a 5-year follow-up, possibly including an identification key (picture, signature) could be used to improve the validity of treatment registers used during community-directed treatment with ivermectin campaigns. Alternatively, biometrics (fingerprint or iris scanning) could be used, as has been demonstrated for other public health applications.^22^

The populations living in areas in need of an alternative treatment strategy for elimination of onchocerciasis has been estimated, using mathematical projections, to be about 14 million in 2015, with the greatest number of individuals located mainly in the Democratic Republic of the Congo, but also in Cameroon, the Central African Republic, and Gabon.^23^ Additional onchocerciasis surveys should be done to refine those estimates. Strategies other than TaNT (such as selective treatment of people infected with onchocerciasis with doxycycline) could be considered in small communities with a very low onchocerciasis prevalence.

Assessment of *L loa* microfilaraemia in an endemic population 18 months after a large-scale treatment with ivermectin showed a notable reduction in the reservoir of *L loa* microfilariae, with microfilarial counts in all previously treated individuals below the level previously associated with neurological SAEs. On the basis of our results, we would argue that individuals treated with ivermectin during the preceding 18 months would not have to be retested for *L loa* microfilaraemia before receiving ivermectin. The reduced number of people in need of retesting would lead to huge cost savings and facilitate reaching programmatic goals for elimination of onchocerciasis in areas that are co-endemic for loiasis.
