# Supplementary material for: Implications for annual retesting after a test-and-not-treat strategy for onchocerciasis elimination in areas co-endemic with Loa loa infection: an observational cohort study
Source: Lancet Infect Dis. 2020 Jan;20(1):102–9. doi: 10.1016/S1473-3099(19)30554-7 (PMC8150319; doi:10.1016/S1473-3099(19)30554-7)
Supplement: Supplementary appendix [file mmc1.pdf]

THE LANCET Infectious Diseases

Supplementary webappendix

This webappendix formed part of the original submission and has been peer reviewed. We post it as supplied by the authors.

Supplement to: Pion SDS, Nana-Djeunga H, Niamsi-Emalio Y, et al. Implications for annual retesting after a test-and-not-treat strategy for onchocerciasis elimination in areas co-endemic with *Loa loa* infection: an observational cohort study. *Lancet Infect Dis* 2019; published online Oct 29.

	TaNT				Gardon et al 1997			
Pre-treatment *L. loa* microfilarial density (mf per mL)	Number of individuals	Arithmetic mean of *L. loa* microfilarial density (mf per mL)		% Reduction	Number of individuals	Arithmetic mean of *L. loa* microfilarial density (mf per mL)		% Reduction
		Baseline	18 months			Baseline	12 months	% Reduction
0	5,649	0	18·8	-	39	0	0·5	-
1–100	3	39·0	0	100·0	41	37·6	9·8	74·0
101–500	274	268·1	255·3	30·2	40	267·0	152·5	42·9
501–2,000	296	1,150·0	448·6	66·9	19	1,029·5	91·6	91·1
2,001–10,000	445	4,943·3	1,295·8	75·4	45	4,459·1	916·0	79·5
10,001–20,000	160	13,946·9	3,464·3	79·4	27	14,277·8	2,906·7	79·6

Table 1. Arithmetic mean of *L. loa* microfilarial density before and after 18 months (present study) and 12 months (Gardon et al 1997) after a first single dose of ivermectin
